# Erratum to: Priority actions for Fusarium head blight resistance in durum wheat: Insights from the wheat initiative

**DOI:** 10.1002/tpg2.20565

**Published:** 2025-01-20

**Authors:** Ambra Viviani, Jemanesh K. Haile, W. G. Dilantha Fernando, Carla Ceoloni, Ljiljana Kuzmanović, Dhondup Lhamo, Yong‐Qiang Gu, Steven S. Xu, Xiwen Cai, Hermann Buerstmayr, Elias M. Elias, Alessia Confortini, Matteo Bozzoli, Gurcharn Singh Brar, Yuefeng Ruan, Samia Berraies, Walid Hamada, Safa Oufensou, Malini Jayawardana, Sean Walkowiak, Salim Bourras, Monika Dayarathne, Julio Isidro y Sánchez, Fiona Doohan, Agata Gadaleta, Ilaria Marcotuli, Xinyao He, Pawan K. Singh, Susanne Dreisigacker, Karim Ammar, Valentyna Klymiuk, Curtis J. Pozniak, Roberto Tuberosa, Marco Maccaferri, Barbara Steiner, Anna Maria Mastrangelo, Luigi Cattivelli

This erratum corrects the following:

The following authors should have been listed as co‐corresponding authors:

Roberto Tuberosa and Marco Maccaferri, Department of Agricultural Sciences, University of Bologna, Bologna, Italy. Email: roberto.tuberosa@unibo.it, marco.maccaferri@unibo.it

Barbara Steiner, Department of Agrobiotechnology Tulln, University of Natural Resources and Life Sciences Vienna, Tulln, Austria. Email: barbara.steiner@boku.ac.at

Anna Maria Mastrangelo, CREA—Research Centre for Cereal and Industrial Crops, Foggia, Italy. Email: annamaria.mastrangelo@crea.gov.it

Luigi Cattivelli, CREA—Research Centre for Genomics and Bioinformatics, Fiorenzuola d'Arda (PC), Italy. Email: luigi.cattivelli@crea.gov.it
